# A motivational interview program for cardiac rehabilitation after acute myocardial infarction: study protocol of a randomized controlled trial in primary healthcare

**DOI:** 10.1186/s12875-022-01721-y

**Published:** 2022-05-06

**Authors:** Rocío Rodríguez-Romero, Carles Falces, Belchin Kostov, Noemí García-Planas, Esther Blat-Guimerà, María C. Alvira-Balada, Mireia López-Poyato, María L. Benito-Serrano, Ingrid Vidiella-Piñol, Juan J. Zamora-Sánchez, Marta Benet, Manuel V. Garnacho-Castaño, Susana Santos-Ruiz, Rosalia Santesmases-Masana, Silvia Roura-Rovira, Jaume Benavent-Areu, Antoni Sisó-Almirall, Luis González-de Paz

**Affiliations:** 1 Consorci d’Atenció Primària de Salut Barcelona Esquerra, Barcelona, Spain; 2 Primary Healthcare Transversal Research Group, Institut d’Investigacions Biomèdiques August Pi i Sunyer (IDIBAPS), Barcelona, Spain; 3 Cardiovascular Institute. Hospital Clínic. Universitat de Barcelona, Barcelona, Spain; 4 Research Group on Atherosclerosis, Coronary Disease and Heart Failure, Institut d’Investigacions Biomèdiques August Pi i Sunyer (IDIBAPS), Barcelona, Spain; 5 Department of Statistics and Operations Research, Universitat Politècnica de Catalunya (UPC), Barcelona, Spain; 6 Institut Català de la Salut, Barcelona, Spain; 7 Research Group on Society, Politics and Inclusive Communities, University of Vic-Central University of Catalonia, Barcelona, Spain; 8 Campus Docent Sant Joan de Déu, C. Miret i Sans, 10-16, 08034, Barcelona, Spain; 9 School of Nursing, Hospital Santa Creu I Sant Pau, Universitat Autònoma de Barcelona, Barcelona, Spain

**Keywords:** Myocardial infarction, Cardiac rehabilitation, Primary healthcare, Motivational interview

## Abstract

**Background:**

Cardiac rehabilitation after acute myocardial infarction permits recovery of the heart function and enables secondary prevention programs in which changes in lifestyle habits are crucial. Cardiac rehabilitation often takes place in hospitals without coordination with primary healthcare and is not focused on individual patient preferences and goals, which is the core of the motivational interview. The objective of this study was to evaluate the efficacy of a cardiac rehabilitation program with a motivational interview in patients discharged from hospital after acute myocardial infarction.

**Methods/design:**

A randomized, non-pharmacological clinical trial in six primary healthcare centers in Barcelona (Spain) will assess whether a tailored cardiac rehabilitation program consisting of four motivational interviews and visits with family physicians, primary healthcare nurses and a cardiologist, coordinated with the reference hospital, results in better cardiac rehabilitation than standard care. A minimum sample of 284 participants requiring cardiac rehabilitation after acute myocardial infarction will be randomized to a cardiac rehabilitation group with a motivational interview program or to standard primary healthcare. The main outcome will be physical function measured by the six-minute walk test, and the secondary outcome will be the effectiveness of secondary prevention: a composite outcome comprising control of blood pressure, cholesterol, diabetes mellitus, smoking and body weight. Results will be evaluated at 1,3 and 6 months.

**Discussion:**

This is the first clinical trial to study the impact of a new primary healthcare cardiac rehabilitation program with motivational interviews for patients discharged from hospital after myocardial infarction. Changes in lifestyles and habits after myocardial infarction are a core element of secondary prevention and require patient-centered care strategies such as motivational interviews. Therefore, this study could clarify the impact of this approach on health indicators, such as functional capacity.

**Trial registration:**

ClinicalTriasl.gov NCT05285969 registered on March 18, 2022.

## Background

Recovery of heart function after acute myocardial infarction (AMI) requires cardiac rehabilitation (CR) to ensure the best possible physical, mental, and social conditions to regain an active life [1, 2]. CR programs have been shown to reduce morbidity and mortality after AMI, and improve the quality of life and psychological wellbeing [3]. However, CR programs are not widely implemented in EU countries: after AMI, not all patients undergo CR, which varies from 3% in Spain to 90% in Lithuania [4].

CR programs include physical training, health education, psychological interventions and control and follow up of risk factors: smoking, hypertension, cholesterol levels, diabetes mellitus, obesity and sedentary lifestyles (physical activity) [4, 5]. CR programs are often divided into three phases, starting after stabilization of AMI: a) in-hospital, b) early outpatient phase, and c) maintenance [6, 7]. The European Society of Cardiology has focused on hospital-based CR programs, but they may also be outpatient led. Delivering hospital CR programs after AMI to all patients has two drawbacks: poor accessibility and delays in starting due to a lack of rooms and healthcare professionals. While the effectiveness of CR is greater if begun early, currently, most patients do not start CR programs one year after AMI, thus increasing the risk of a worse outcome [8]. A European study showed that only 45% of patients discharged from hospital after AMI with or without revascularization, were referred for CR and only 34% participated [9]. A possible solution to overcoming the barriers to CR programs would be to integrate CR into primary healthcare (PHC).

PHC centers facilitate health care in the community, and citizens have an assigned family physician and PHC nurse [10]. PHC health professionals coordinate with other healthcare professionals, such as cardiologists, and other healthcare settings, including hospitals [11, 12]. PHC services also include home-care programs for patients unable to attend the PHC center due to health problems or disability. PHC physicians and nurses are well positioned to care for patients requiring CR after AMI, because the main objectives are to control risk factors, improve patient self-management and decision-making in diet, exercise routines and weight control, etc. A review and a meta-analysis concluded there were no differences between CR at home or in hospitals with respect to mortality, reinfarction, revascularization, hospitalization, and exercise capacity [13, 14].

In CR, person-centered care is essential, including consideration of patients’ goals, values, previous routines, and environment [15, 16]. This approach requires communication skills, such as motivational interviews (MI). MI is a collaborative, goal-oriented style of communication with particular attention paid to the language of change [17]. In PHC, MI make it possible to establish common objectives that can be monitored agreed between patients and health professionals and which encourages motivation to change by exploring and solving patients’ ambivalence [18]. MI and CR are more effective in the early stages of the disease, for example, after AMI, when the patient is more likely to initiate lifestyle changes [19, 20]. Two systematic reviews showed that MI improved self-care in patients [21, 22] with heart failure. However, the effect of MI combined with a CR program after AMI is unclear. Therefore, in this protocol we plan to study the efficacy of CR using MI compared with the current PHC standard of care after hospital discharge for AMI.

## Methods/design

### Main objective

The main objective of the study is to evaluate the efficacy in functional capacity, lifestyle indicators and psychological wellbeing of a new CR program with MI carried out entirely in primary healthcare in patients discharged from hospital after AMI.

### Secondary objectives and hypothesis

(i) To compare improvements in functional capacity and changes in risk factors (secondary prevention). (ii) To evaluate the impact of the CR program according to adherence to therapy (drug treatment and physical activity program) and health service use and (iii) to evaluate the efficacy of the CR program based on psychological factors and quality of life after AMI.

The hypothesis of the study is that a PHC CR program with MI after AMI has a positive impact on functional and psychological wellbeing and quality of life compared to standard care.

### Trial design and study setting

**Fig. 1:**
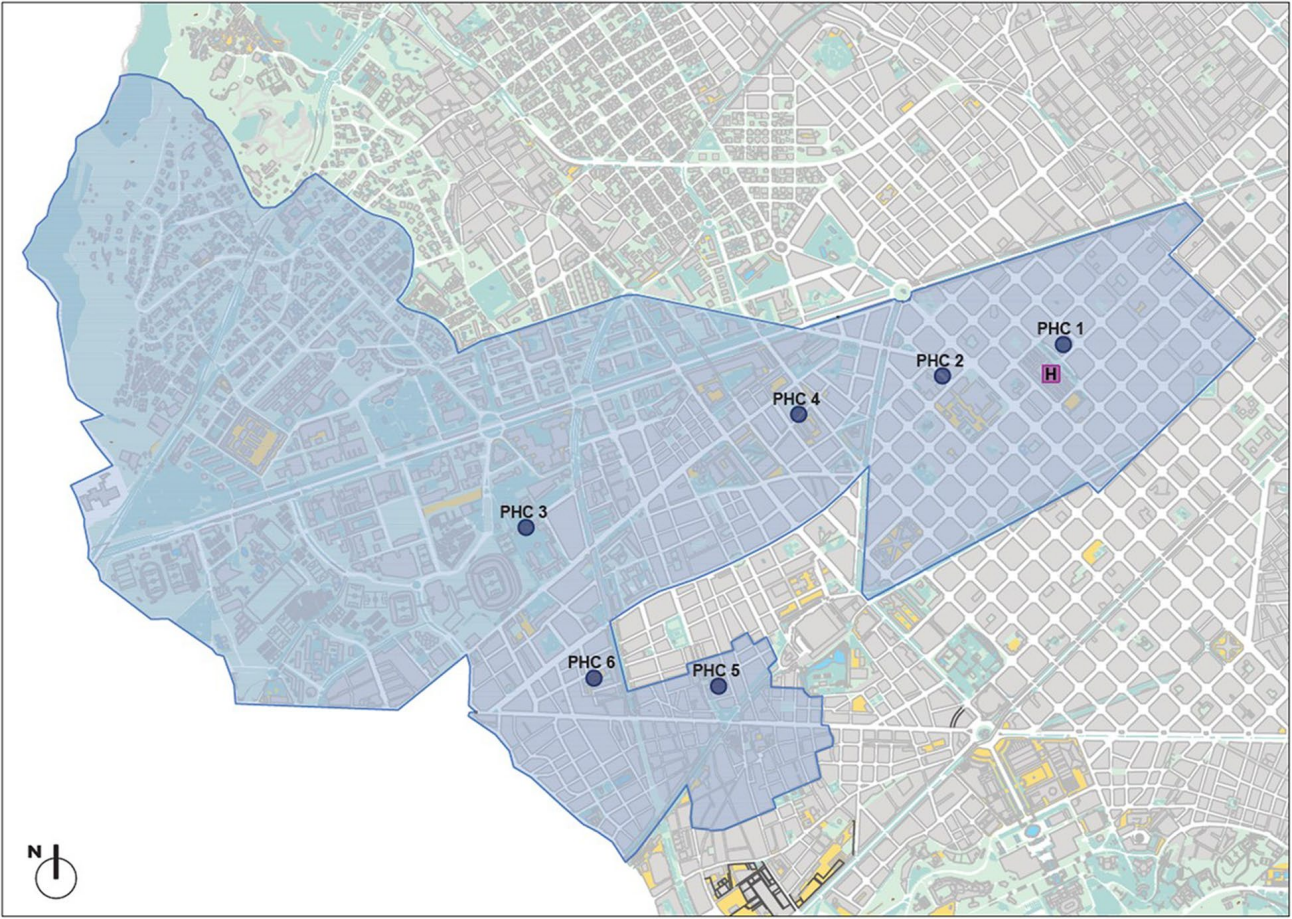
Partial map of Barcelona (1:30,000). Area of influence of the six PHC (blue), and the Hospital Clinic of Barcelona. The map was modified from the Cartographic and Geological Institute of Catalonia, which gave permission to reuse their data and content [36]

This will be a randomized controlled trial with two arms: a PHC CR program including MI (Intervention group) versus PHC standard care (Control group). The study will be carried out in seven primary healthcare areas in Barcelona city with six assigned PHC centers, including family physicians, nurses and social workers. The six PHC provide healthcare to 187, 223 people [23] and coordinate actions with the Hospital Clinic of Barcelona, the public high-complexity reference hospital, with an assigned population of 540,000 [24]. Figure 1 shows a map of the area of influence of the PHCs and the location of the hospital. The trial was prospectively registered (before participant recruitment) on ClinicalTrials.gov (NCT05285969) on March 18, 2022.

### Participants and eligibility criteria

Potential participants will be PHC patients admitted to the reference hospital due to acute coronary syndrome (diagnostic codes ICD-10: I20-I22) or post-unscheduled cardiac revascularization surgery (code ICD-10: 021x) and discharged to home in the area of the six PHCs. Inclusion criteria will be age > 18 years, indication for CR and voluntary participation. Exclusion criteria will be: (1) acute aortic disease, severe pulmonary hypertension, uncontrolled arrhythmia, decompensated heart failure or significant valvular or congenital heart disease, (2) heart valve and/or interventricular septum surgery, (3) diseases that prevent exercise, (4) osteoarticular diseases that severely limit exercise, (5) severe mental disorder (i.e. schizophrenia, bipolar disorder, major depression or autism, and severe forms of other disorders), (6) cognitive disability, (7) problems of verbal communication and, (8) inclusion in a hospital CR program.

### Intervention group

CR with MI will be structured in four sessions, with an optional fifth session, in the six months after discharge. The methodology of the MI sessions will follow the four-phase logical sequence of MI proposed by Rollnick and Millner 1) engage in collaborative relationships, 2) focus on a particular change, 3) evoke intrinsic motivations for change, and 4) plan an immediate step for change [25]. MI will be administered by nurses trained through a certified MI course, who will be offered additional support and counseling. Collaborators will meet at least once a month to standardize the intervention and follow up of issues regarding MI. Each MI session will have defined contents and objectives to ensure homogeneity. The objectives of CR and secondary prevention will be introduced from the first session, (i.e., to increase participation in activities of daily living and self-care and follow recommendations on safe physical activity). Table 1 describes the MI program and the content of each session. Interviewers will collaborate in the coordination of care, ensuring continuity and communication between PHC family physicians, nurses, and the cardiologist).

**Table 1:** Description of the intervention, content and aims of MI sessions

Session	Schedule and location	Duration	Aims	Content Description
#1	72 h Home	45 min	Relationship between patient and interviewer. Promote involvement of participant in CR	Presentation. General information on the program. Examine experiences, knowledge, motivation, self-confidence and expectations of CR and prevention
#2	2nd week PHC	20–30 min	Strengthen commitment and link with professional. Increase self-efficacy. Strengthen motivation	Identify specific objectives of patient to adjust to the CR plan. Collaborative work on personalized CR. Support and active listening to participant concerns. Examine participant resources
#3	5th week PHC	20–30 min	Maintenance therapeutic link. Empower participants. Increase self-efficacy. Strengthen motivation	Collaborative work. Support and active listening to patient concerns and adaptation of RC. Examine resources, barriers, and facilitators of patients. Manage discrepancies and resolving ambivalence. Discourse of maintenance
#4	8th week PHC	20–30 min	Empower patients. Increase self-efficacy. Reinforce motivation to maintain CR plan and prevention	Collaborative work in adapting the CR plan of persons at home. Support and active listening to concerns for maintenance. Examine resources, barriers and facilitators of patients about the CR plan at home and in the community. Discrepancy management. Resolve ambivalence
#5 (Optional)	13th week PHC	2–30 min	Link maintenance. Empower participants. Increase self-efficacy	This session can be programmed according to the criterion of the interviewer and depending on the participant's status. Tasks: Collaborative work. Support and active listening to concerns. Adaptation to the CR plan. Examine resources, barriers and facilitators. Discrepancy management. Resolve ambivalence. Maintenance discourse management

### Standard care group

To standardize comparison with the MI group, all control group patients will receive a kit with information about the actions and procedures to follow (diet, physical activity, smoking cessation, and other recommendations on secondary prevention) and the home physical activity program. Home exercises will be adapted from cardiology guidelines from the United States, Canada and Europe [26]. The collection of data, analytical samples, and information (questionnaires, scales, and clinical information) will be the same as for the intervention group. A collaborating researcher will contact patients by telephone beforehand. Table 2 shows the SPIRIT chart [27], describing the schedule of enrollment, interventions, and assessments.

**Table 2:** Schedule of procedures, template from the SPIRIT check list. **v**_9_* I an optional visit, according to participant status (see Table 1)

			**Week**													
			**0**	**1**			**2**	**4**	**5**		**8**	**12**	**13**		**23**	**24**
			**Visit**													
**Activity/Task**	**Group**	**Agent, health care professional**	**v-**_**1**_	**v**_**0**_	**v**_**1**_	**v**_**2**_	**v**_**3**_	**v**_**4**_	**v**_**5**_	**v**_**6**_	**v**_**7**_	**v**_**8**_	**v**_9_^*^	**v**_**9**_	**v**_**10**_	**v**_**11**_
Eligibility screen	Both	Research collaborator	x													
Informed consent	Both	Research collaborator	x													
Allocation	Both	Research collaborator		x												
Data collection of participant status	Both	Research collaborator			x			x				x			x	
Motivational interview	Intervention	Research collaborator			x		x		x		x		x			
Follow up: family physician	Both	Family physician				x			x					x		x
Follow up: PHC nurse	Both	PHC nurse				x			x					x		x
Six-minute walk test	Both	Research collaborator						x							x	
Follow up: cardiologist	Both	Cardiologist of the PHC area								x						
Blood analysis	Both	Research collaborator										x			x	

### Strategies to improve adherence to the protocol

To maximize adherence, study collaborators will send reminders of data collection sessions and visits by phone. If patients do not attend, they will be contacted again to avoid losses. Sessions will take place at the initial product dispensing and each study visit thereafter. The only criteria for discontinuing the intervention will be hospitalization due to worsening status. All concomitant care and interventions for health reasons are permitted during the trial.

### Main outcome

#### Physical functional capacity

Improvement in aerobic capacity and resistance, measured by the six-minute walk test [28]

### Secondary outcomes

#### Effectiveness of secondary prevention

A composite variable that groups secondary prevention measures: BP (values < 140/90 mmHg), cholesterol (c-LDL < 70 mg/dL), diabetes mellitus (plasma glycosylated hemoglobin < 7%), absolute cessation or no initiation of smoking and weight (body mass index in the range of 18.5-25 kg/m2).

#### Psychological status and quality of life

Measured by the Psychological General Well-Being Index (PGWBI) [29] and the generic SF-12 [30].

#### Other variables and factors

Variables are described in Table 3 and tests or instruments and their characteristics in Table 4, including sociodemographic and household characteristics, clinical status, use of health services, disease management, and lifestyle habits and psychological and emotional status.

**Table 3:** Description of variables and operationalization

Group	Name of variable/Factor	Operationalization
**Sociodemographic and household characteristics**	Age	Date of birth
	Sex	Male/female/non-binary
	Educational level	Primary education not completed/Primary education/Secondary Education/Vocational studies/University degree or higher
	Main source of income	Employment/unemployment allowance/disability/retirement pension/social aid (financial welfare benefits)
	Household income	Total €
	Profession/job	Job definition
	Residents at home	Number of residents living at household
**Clinical status**	Anthropometry	Weight (kg), Height (cm) and Waist diameter (cm)
	Body Mass Index	Categorized as normal weight, overweight, obesity and severe obesity
	Blood Pressure	Systolic blood pressure/Diastolic blood pressure (mmHg)
	Diagnoses	Diabetes Mellitus, dyslipidemia, hypertension, all as registered in the electronic health record
	Severity of comorbidity	Results of Charlson Comorbidity index
	Cardiac function	Echocardiographic results at discharge, and ejection fraction,
	Early risk stratification	Results of Canadian Acute Coronary Syndrome Score C-ACS
	Blood analysis results	Erythrocyte counts, biochemistry, lipid and hepatic profile, HbA1c (%), Glucose, mg/dL, Total cholesterol, mg/dL, Cholesterol- HDL, mg/dL, Cholesterol-LDL, mg/dL, triglycerides, mg/dL
	Severity of comorbidity	Charlson Comorbidity Index. (0 to 100)
	Sexual functionality	Alterations due to heart disease
	Active pharmacological prescription	Antihypertensives, Antidiabetics, Antithrombotic, Cholesterol-lowering drugs, Gastric protectors, Thyroid hormone therapy, Antidepressants, Bronchodilators, Opioid analgesics, Other
**Use of Health Services**	Visits to health care centers	Hospitalizations in last 6 months/Emergency service and hospital readmission in last 6 months/PHC nurse visits in last 6 months/PHC family physician visits in last 6 months/Cardiologist visits in last 6 months
**Disease management and lifestyle habits**	Adherence to drug treatment	Results of Morinsky-Green questionnaire
	Adherence to physical activity program	Yes/No
	Alcohol consumed	Systematic Interview of Alcohol Consumption (ISCA)
	Physical activity	IPAQ questionnaire
	Smoking habit	Non-smoker/smoker/ex-smoker (1 year not smoking)
	Patient activation (self-efficacy)	13-item Patient Activation Measure (PAM-13)
**Psychological and emotional status**	Depressive symptoms	Patient Health Questionnaire. PHQ-9
	Cognitive dysfunction screening	Montreal Cognitive Assessment
	Perceived functional social support	DUKE UNC-11 questionnaire
	Depression/anxiety	Hospital Anxiety and Depression Scale (HADS)

**Table 4:** Questionnaires and instrument characteristics planned for use in the trial. ISCA: systematic interview of alcohol consumption

Test/Instrument	Variable/ object of measurement	Characteristics/information
**Morinsky-Green** [37, 38]	Adherence of drug treatment	**Structure:** Self-administered, 4 items with a dichotomous answer YES/NO
		**Interpretation**: Informs about the causes of non-compliance. Compliance is considered if 4 questions are correct (No/Yes/No/No)
**Systematic Interview of Alcohol Consumption. ISCA** [39]	Alcohol consumed	**Structure:** Professional administered, 3 items, allows examination of the amount of alcohol consumed, the frequency, and the variation between work-days and weekends in Standard Drink Units
		**Interpretation:** High risk alcohol consumption is considered if > 28 units in men or > 17 units in women
**Canadian Acute Coronary Syndrome Score. C-ACS** [40]	Early risk stratification	**Structure**: Score ranges from 0 to 4 according 4 items scored with 1 if: age ≥ 75 years, Killip class > 1, systolic blood pressure < 100 mm Hg, and heart rate > 100 bpm
		**Interpretation:** Short- and long-term mortality in patients with acute coronary syndrome
**Charlson Comorbidity** [41]	Severity of comorbidity	**Structure:** Professional administered, 19 diseases rated from 1 to 6, total score between 0–37 points. From 50 years of age, one point is added for each decade
		**Interpretation:** Higher scores indicate a more severe condition and worse prognosis, can be categorized to no comorbidity, low comorbidity, and high comorbidity
**Short Form Health Survey. SF-12** [30, 42]	Health-related quality-of-life	**Structure:** Self-administered, 12 items with Likert-type ratings, ranging from 3–6 points
		**Interpretation:** Results can be standardized to a 0–100 range and then summarized in two dimensions: physical component summary and mental component summary
**DUKE UNC-11** [43, 44]	Perceived functional social support	**Structure:** Self-administered, 11 items with Likert-type ratings ranging 1 to 5. Scores range from 11 to 55 points
		**Interpretation:** The higher the average score, the greater the perceived social support
**Montreal Cognitive Assessment** [45, 46]	Cognitive dysfunction screening	**Structure:** Professional administered consisting of 12 tasks of the following domains: visuospatial/executive (5 points), naming (3 points), attention (6 points), language (3 points), abstraction (2 points), delayed recall (5 points), orientation (6 points). Total scores range between 0 and 30
		**Interpretation:** A score < 26 might indicate cognitive impairment
**Patient Health Questionnaire. PHQ-9** [47]	Depressive symptoms	**Structure:** Self-administered, 9 items for screening, diagnosing, monitoring and measuring the severity of depression
		**Interpretation:** Scores and cut-points: 5, 10, 15, and 20 represent for mild, moderate, moderately severe, and severe depression respectively
**Hospital Anxiety and Depression Scale (HADS)** [48, 49]	Emotional distress (anxiety and depression)	**Structure:** Self-administered, 14 items (7 anxiety and 7 depression), with Likert-type ratings ranging 1 to 4. Total ranging from 0 to 21
		**Interpretation:** A total subscale score of > 8 points in each of the sub-scales might indicate depression or anxiety
**International Physical Activity Questionnaires (IPAQ)** [50, 51]	Frequency, duration and intensity of activity	**Structure**: Self-administered, 7 questions self-completed. Evaluates: intensity (low, moderate, or vigorous), frequency (days for week) and duration (time for day)
		**Interpretation:** Classifies the level of activity in three categories: low, moderate, and high. Permits individual results to be converted into METs
**Six-minute walk test** [28]	Aerobic capacity and endurance	**Structure:** Sub-maximal exercise test used to assess aerobic capacity and endurance. The distance covered over a time of 6 min is used as the outcome to compare changes in performance capacity
		**Interpretation:** Allows results to be converted from meters to METs. An increase in the distance walked indicates improvement in physical function
**The 13-item Patient Activation Measure (PAM-13)** [52, 53]	Patient activation (self-efficacy)	**Structure**: Self-administered. 13 items 4-point Likert scale. Total scores range from 0 to 100
		**Interpretation**: Scores are converted into Rasch units defining 4 stages of activation: 1. Believes active role is important; 2. Confidence and knowledge to take action 3. Taking action; 4. Staying the course under stress
**Psychological General Well-Being Index (PGWBI)** [29]	Psychological status and quality of life	**Structure:** Self-administered, 22 items, with 6-point Likert scale, and five dimensions: anxiety, depression, positive mood, vitality, self-control and general health. Total score range is from 0–110
		**Interpretation:** Lower scores indicate more severe distress. The three levels of distress are scored as: 0 to 60 reflect severe distress; 61 to 72 moderate distress; and 73 to 110 positive well-being

#### Participant timeline

**Fig. 2:**
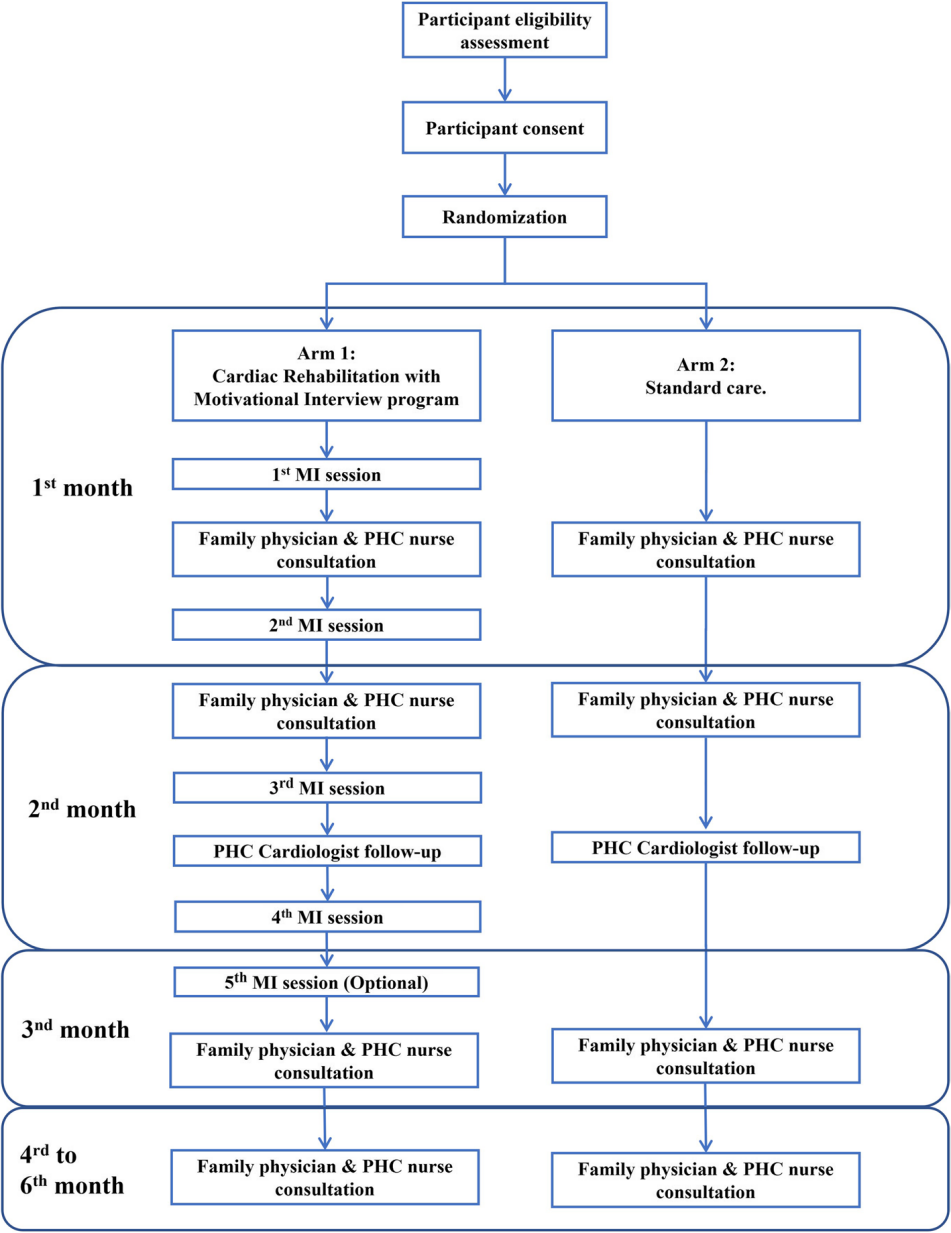
Study flowchart

Patients meeting the inclusion criteria will be asked to participate during hospital admission. Potential participants will be given written and verbal information about the study and, if they agree to participate, will be asked to complete the informed consent document. The study coordinator will assign patients to the study groups using a previously-generated blinded random sequence. Participants will be contacted to specify the date of the baseline data collection visit. Sociodemographic and baseline characteristics will be evaluated at the beginning of the study. Follow-up evaluations will be made at 1, 3 and 6 months. Baseline and follow-up evaluations will be made by researchers unaware of the group to which each patient is assigned. Figure 2 shows the study flowchart and timeline.

### Sample size calculation

The six-minute walk test is sensitive and specific in measuring changes in functional capacity. Evidence shows the minimum clinically-relevant difference is 30 m [31, 32]. Accepting an alpha risk of 0.05 and a beta risk of 0.2 in a bilateral contrast, with a standard deviation of 80 m in the six-minute walk test, 284 participants (142 in the intervention group and 142 in the control group) will be required to detect differences of ≥ 30 m. To achieve adequate participant enrollment and reach the minimum sample size, we analyzed the incidence of new AMI in the six PHC areas and foresee that three years will be sufficient to reach the minimum sample size.

### Randomization

A randomization sequence will be generated and participants will be assigned using a centralized method with hidden assignation. Only the nurses who carry out the MI will be aware of the participants in the MI group. The assessments at 1, 3 and 6 months will be made by an assessor blinded to the group assignment. The study coordinator will record whether they were informed of the assignment of participants.

### Data management and monitoring

All study information will be saved securely, and all participant information will be stored using an electronic secure server system with limited access. The information will be identified by a coded identification number to ensure confidentiality. All records with names or other personal identifiers (like locator forms and informed consent forms) will be stored separately from study registers and identified by a coded number. The main database will be protected with a password-protected system.

Questionnaires will be stored after informed consent. Follow-up questionnaires will be collected at 3 and 6 months after the baseline questionnaire. For follow-up visits (control blood tests and administration of questionnaires), a researcher will make an appointment with patients by phone and provide the dates of interviews.

Data management and monitoring will follow the pre-planning foreseen in the monitoring plan. A data monitoring committee (principal investigator, statistician and a collaborator from each PHC) will ensure the integrity of data recording.

### Statistical methods

Outcomes will be evaluated at 1, 3 and 6 months. Participant characteristics will be described using central tendency measures: mean or median and variability: standard deviation or interquartile range for continuous variables, and frequencies and percentages for categorical variables. The results of the physical functional capacity and the six-minute walk test will be transformed into units of metabolic equivalent of task (MET) using the equation and conversion table of the American College of Sports Medicine [33]. Between-group differences will be studied using the Student’s t-test for two samples. The magnitude of the effect will be calculated using Cohen's D. The effect of the intervention or standard care will be calculated using the Student’s t-test or Mcnemar’s test for paired samples. A mixed linear regression model will be used to evaluate trends in each arm, adjusting for variables of interest. For the inference analysis, co-variables that correlate (sociodemographic factors) will be used and adjusted analyzes made. In the comparison of multiple hypotheses, adjustment of the level of statistical significance (α = 0.05) will be used to avoid type I errors. All analyzes will made per protocol and intention-to-treat. The analysis will be made using R v3.5.2 [34].

## Discussion

This protocol aims to study the effectiveness of a post-AMI CR program with MI in PHC. CR will be carried out in PHC centers and may be innovative in allowing patients to access CR.

No differences in mortality, reinfarction, revascularization, hospitalization, and exercise between CR at home or in hospitals have been shown [13]. However, there is a lack of programs and research specifying CR programs in PHC, and no indicators have assessed standard care. As no program has been designed specifically in Spain, if the results of this trial are as expected, this PHC-based program could increase participation in CR post-AMI programs. Research on non-hospital-based CR programs has a poor level of methodological reporting, with details of interventions often poorly reported. Therefore, to achieve sufficient quality, we have followed the seven core recommendations of the UK CR standards [35]. The fourth component refers to the assessment of patient needs which, in the PHC context, refers directly to a patient-centered approach. In our CR program, the patient-centered approach and the patient’s self-perceived objectives will be covered by the MI component which, at the same time is the core component of the whole program. Our perspective is that MI rehabilitation will be effective only when placing the patient as the individual at the center of the whole CR program.

### Limitations of the study

The study will be carried out in patients from a single, urban hospital. This may limit the generalizability of the results to semi-urban or rural areas. Second, the complete blinding of participants to their group assignment may not be guaranteed because patients may discover they have been assigned to the control group. However, this issue is common in MI research. Finally, the duration of the follow-up will not allow study of the long-term effects, although a subsequent study with a cohort design is possible. In addition, the proposed study is restricted to the improvement in the first six months of outpatient treatment of CR.

## Data Availability

The study steering committee will accept requests for data sharing once the study is completed. The steering committee will evaluate the scientific soundness of each proposed project and will grant access whenever projects seem scientifically sound. All data sharing will apply to national and international legislation, rules, and other regulations by regional or national authorities. Applications for data require a formal application and will be decided upon by the board of the scientific group.

## Abbreviations

AMI: Acute myocardial infarction
BP: Blood pressure
CR: Cardiac rehabilitation
CRP: Cardiac rehabilitation programs
CVD: Cerebrovascular disease
DM: Diabetes mellitus
HF: Heart failure
LDL: Low-density lipids
MI: Motivational interviewing
PHC: Primary healthcare
RCT: Randomized clinical trial
